# Hyaluronic Acid-Silver Nanocomposites and Their Biomedical Applications: A Review

**DOI:** 10.3390/ma15010234

**Published:** 2021-12-29

**Authors:** Joanna Dulińska-Litewka, Kacper Dykas, Dominik Felkle, Karolina Karnas, Gohar Khachatryan, Anna Karewicz

**Affiliations:** 1 Chair of Medical Biochemistry, Jagiellonian University Medical College, Mikołaja Kopernika Street 7C, 31-034 Krakow, Poland; kacper-4444@wp.pl (K.D.); dominikfelkle@gmail.com (D.F.); karo.karnas@gmail.com (K.K.); 2 Department of Chemistry, Jagiellonian University, Gronostajowa 2, 30-387 Cracow, Poland; karewicz@chemia.uj.edu.pl; 3 Faculty of Food Technology, University of Agriculture in Cracow, Balicka Street 122, 30-149 Krakow, Poland; gohar.khachatryan@urk.edu.pl

**Keywords:** silver nanoparticles, hyaluronic acid, biopolymers, nanocomposites, polysaccharides

## Abstract

For the last years scientific community has witnessed a rapid development of novel types of biomaterials, which properties made them applicable in numerous fields of medicine. Although nanosilver, well-known for its antimicrobial, anti-angiogenic, anti-inflammatory and anticancer activities, as well as hyaluronic acid, a natural polysaccharide playing a vital role in the modulation of tissue repair, signal transduction, angiogenesis, cell motility and cancer metastasis, are both thoroughly described in the literature, their complexes are still a novel topic. In this review we introduce the most recent research about the synthesis, properties, and potential applications of HA-nanosilver composites. We also make an attempt to explain the variety of mechanisms involved in their action. Finally, we present biocompatible and biodegradable complexes with bactericidal activity and low cytotoxicity, which properties suggest their suitability for the prophylaxis and therapy of chronic wounds, as well as analgetic therapies, anticancer strategies and the detection of chemical substances and malignant cells. Cited studies reveal that the usage of hyaluronic acid-silver nanocomposites appears to be efficient and safe in clinical practice.

## 1. Introduction

Nanotechnology is a rapidly developing branch of science which has a potential to influence all aspects of our lives, including medicine. Nanoparticles are objects with dimensions (or at least one dimension) that are smaller than 100 nm [1], characterised by unique physicochemical, functional, and biological properties [2,3]. They are used in pharmacy to improve drugs’ biodistribution, or to deliver them directly to the specific places or to the specific cells. Their applications in medicine are numerous, including tissue engineering, hyperthermia, biosensors, and laboratory diagnostics. Some nanoparticles are known to exhibit antibacterial properties, so there is nothing unexpected in the fact that they found application in production of wound dressings and medical containers [4].

Nanoparticles of metals such as palladium (Pd), rhenium (Re), osmium (Os), iridium (Ir), gold (Au), platinum (Pt), and especially silver (Ag), play significant role in nanomedicine [5,6]. Silver nanoparticles are well known to exhibit strong antimicrobial properties, thus they are broadly used as an active component of antiseptic materials (wound dressings, bandages [7,8,9]); some of them are already commercially available [1]. But the possibilities of applying silver nanoparticles in biomedicine have not been yet fully exploited. New horizons for them emerge when we consider combining them with biocompatible polymers.

Polymeric materials may be used either as matrices for biologically active nanoparticulate systems or as a coating in order to modify their physicochemical properties [10], biocompatibility or interactions with cells or tissues. Metal nanoparticles, on the other hand, are often responsible for improved mechanical properties of the polymeric materials. Biocompatible polymers can be divided into two groups, namely (1) synthetic (e.g., poly (vinyl alcohol), poly (vinylpyrrolidone)), and (2) natural polymers, isolated from plants or other living organisms. The latter, due to their origin, are more compatible with human body and often characterized by advantageous properties, such as absence of toxic metabolites, or ability to form hydrogels. Naturally occurring polymers, such as hyaluronic acid (HA), agar, alginate, pectin, starch, carrageenans, chitin, cellulose and their derivatives (chitosan, carboxymethylcellulose) are characterized by high biocompatibility and unique biological activity [1,11] and thus can bring new opportunities into various medical applications.

**Figure 1:**
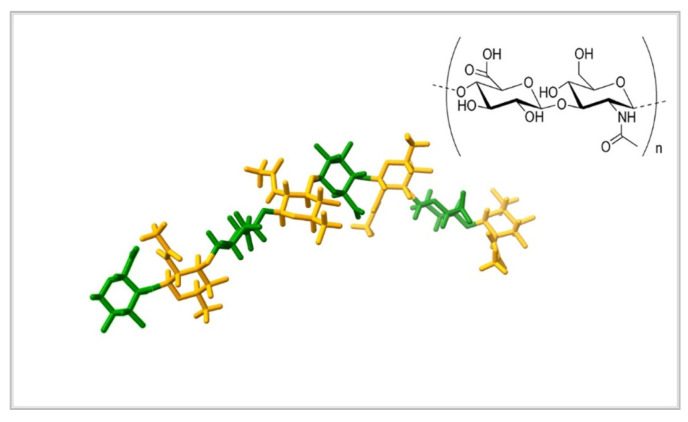
The structure of the hyaluronic acid. The polymer consists of α-D-glucopyranuronic acid (green) and 2-acetamido-2-deoxy-β-D-glucopyranose (orange) linked by glycosidic bonds. The polymer can reach extremely large volumes by gathering molecules of water. Source: Protein Data Bank [16]. Structural formula reprinted from Reference [17], based on CC BY license.

Among naturally occurring polysaccharides hyaluronic acid (HA) has a special place, mainly due to its unique biological activity. HA is a negatively charged, linear, polysaccharide, composed of D-glucuronic acid and N-acetyl-D-glucosamine, which are linked by β-1,3- and β-1,4-glycosidic bonds (Figure 1). It belongs to the class of glycosaminoglycans (GAGs), which can be found in the epithelial, connective, and nervous tissues of vertebrates. In a way HA is unique among GAGs as, unlike the rest, it is not sulphated and can reach very high molecular weight (10^8^ Da). HA constitutes the major GAG component of the extracellular matrix (ECM) [12]. At physiological pH all of its carboxylic groups are dissociated and form salts, hyaluronates, which are highly hydrophilic and surrendered by water molecules. These molecules interact with carboxyl and acetamido groups of HA via hydrogen bonds, stabilizing its structure in the form of a single-strand left-handed helix with two disaccharide residues per turn (two-fold helix) [13]. These two-fold helixes organize into a ß-sheet tertiary structure, which leads to the aggregation of polymeric chains into an extended hydrogel network. As a result, HA can absorb a lot of water, increasing its dry volume by up to 1000 times, and forming viscous hydrogels. A higher molecular weight, as well as higher concentration of the polymer, both lead to the increased viscosity and viscoelasticity of HA solutions/gels [14]. As a consequence of weakened interactions between HA chains, an increase in pH, ionic strength and temperature causes a decrease in the viscosity of HA solutions. In pH lower than 4 or higher than 11 HA degrades by hydrolysis.

HA is widely present in various organisms (humans, animals, bacteria, algae, yeast). As much as 50% of all the HA resides in the skin (the dermis and the epidermis). It can act as passive structural macromolecule (modulation of tissue hydration, osmotic balance and physical properties of ECM) or as a signaling molecule. It was therefore confirmed to play an important role in modulating various biological processes, including tissue repair, signal transduction, angiogenesis, cell motility, and cancer metastasis [15]. CD44 (cluster determinant 44), which is considered the main cell surface receptor for HA, is overexpressed in many cancers, thus HA may be used for targeted delivery of anti-cancer drugs.

Illustration created using UCSF ChimeraX (version 1.2.5.), developed by the Resource for Biocomputing, Visualization, and Informatics at the University of California, San Francisco, with support from National Institutes of Health R01-GM129325 and the Office of Cyber Infrastructure and Computational Biology, National Institute of Allergy and Infectious Diseases [18].

This review gives an overview of the current state of art in the field of the composites of silver nanoparticles and biopolymers based on hyaluronic acid. It presents the methods of synthesis of such composites, their physicochemical and biological properties, and discusses medical implementation of these materials.

## 2. Synthesis

**Figure 2:**
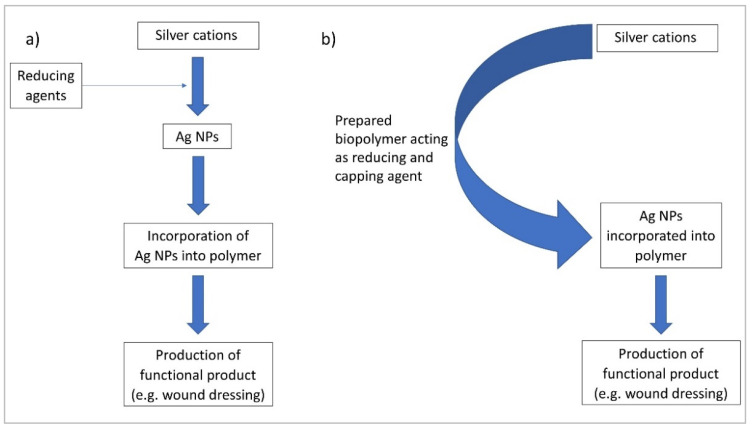
Examples of Ag NPs biopolymer synthesis pathways: (**a**) a typical approach and (**b**) using the biopolymer as reducing agent.

To obtain the composites of silver particles and polymers two main steps are needed: (1) the synthesis of Ag nanoparticles (Ag NPs) and (2) introduction of the polymer chains on the Ag NPs surface or incorporation of Ag NPs into the polymer matrix (Figure 2a).

Typically, Ag NPs are obtained by reduction of silver cations. Silver salts (e.g., silver nitrate) are used as a source of silver ions, while ascorbic acid [19,20,21], trisodium citrate [22,23,24,25] and sodium potassium tartrate [25] are most often applied as reducing agents. The second step, namely coating Ag NPs with biopolymers or embedding them into the polymeric matrix, depends on the composite composition and design, and may be achieved using various approaches and reaction conditions. One of the frequently exploited solutions is to embed Ag NPs into the biopolymer structure in the course of the silver ions reduction process [19,20,21,26].

An interesting approach, proposed in the literature, is to modify the groups already present in the polymeric chain (e.g., hyaluronic acid (HA), corn silk extract, pectin) changing them into the effective reducing agents for silver cations [27,28,29] (Figure 2b). Such methods will probably become more popular in the future, because they minimize the amount of the toxic reagents used and therefore represent a “green chemistry” approach, which takes into account the environmental impact of the whole synthetic process.

**Figure 3:**
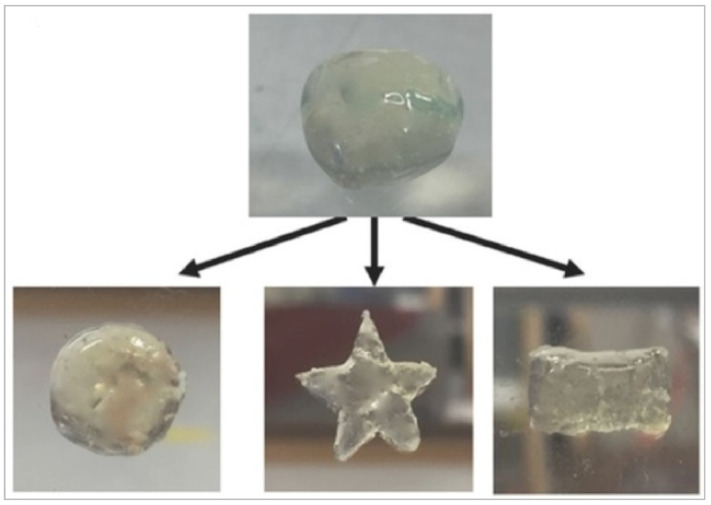
Example of HA-Ag NPs hydrogel and illustration of the possibility of its precise fitting to wounded site. Reprinted from Reference [33] with permission from John Wiley and Sons.

Depending on the polymer used, the composite form and the technique used to produce it, the resulting material may be tailored to the requirements of the application in order to obtain the functional product. In the case of wound dressings, where such composites are applied most often, the methods such as electrospinning [24,28,29,30] the layer-by-layer process [20,31,32] or the drying of the Ag NPs suspension on the polyethylene membrane [26] are used. Hydrogels are also excellent materials for wound dressings; therefore, Ag NPs composite hydrogel materials were also developed [27,33] as they fill wounded site and do not need to be cut into prespecified shape (Figure 3).

Electrospinning is a process in which electric force is used to form fibres from polyelectrolytes. Biopolymers can be easily processed using this method, because it does not require aggressive conditions and is well-suited for macromolecules. In medicine, it is used in tissue engineering, drug delivery and to produce nanofibrous mats used as wound dressings with favourable properties [28].

Layer-by-layer (LbL) method allows creating thin polymeric films by a subsequent deposition of oppositely charged macromolecules. The number of layers strongly influences the mechanical features of the film, so they can be changed in a simple way by adjusting the time of the process. In the case of HA and Ag NPs, the two-component LbL composite is impossible to obtain due to negative charge of HA and no charge of Ag NPs, but the addition of positively charged polymer (e.g., chitosan) resulted in the formation of functional wound dressing [32].

## 3. Physicochemical Properties of HA/AgNPs Biopolymers

Considered in the article polymers were hydrophilic (based on the water contact angle which oscillates depending on the used material in range of almost 0° (for the top layer of polymers synthetized by Vale et al. [31] to 64,2° [29]) which is a favorable behavior in the case of wound dressing. Also, wettability and swelling properties were commonly measured, showing significant ability of water uptake [19,23,31] indicating usefulness of the materials in absorbing the exudation. The nanocomposites exhibited relatively high resistance to degradation mediated via enzymes (e.g., lysozyme [30]). Moreover, the polymers were unaffected by disintegration in physiological-like conditions [20], but in the case of one substance the degradation was quite high [19]. Quantity of the silver released from the biopolymer fluctuated in studies [20,21,28,33], but generally relied on the application of the polymer. It is worth to mention that one composite [28] can adapt the release of antibacterial ions in response to bacterial activity measured by hyaluronidase presence. Thermogravimetric analysis (TGA) results for these biocomposites were similar, showing low weight loss to the temperature of ~250 °C and further rapid decline of mass which stops at about ~400 °C [19,23,27,29]. The exact thermal degradation profile depends on the other substrates in the polymer structure but the share of HA in it is the most marked. Comparison of Young’s modulus of assessed materials shows vide range of values [28,31,33], suggesting that stiffness of the biocomposites is predominantly dependent on the synthesis method and other substances forming polymer. Elongation at break of discussed polymers is relatively low (e.g., about 3–4% for material proposed by Abdel-Mohsen et al. [27]. Despite the results of tensile testing indicating meagre mechanical properties, the HA/AgNPs biopolymers still might serve an important role concerning their potential medical application where this characteristic is not a significant drawback. Their combination with other materials in functional product could also solve this disadvantage.

Aforementioned substances manifest many physical properties favorable in designed applications. Biomaterial synthetized by Liyan et al. [33] achieved proportion of storage (elastic) modulus (G′) and loss (viscous) modulus (G″) characteristic for hydrogels (G′ > G″), which enables the possibility to freely form any shape—it is important in creation of perfectly fitted wound dressing. Design of biocomposite made of Pluronics blends, corn silk extract, HA and AgNPs, that has gelation temperature (measured as the point of temperature when G′ modulus becomes higher than G″ modulus) similar to physiological body temperature [27] is even more promising as such substance can be injected into wounded site and then creates fitted dressing. Indeed, the hydrogel exposed favorable for substances injected by needle viscosity behavior with shear thinning effect. Also, the bone filler biocomposite might be easily injected after suspension in polysaccharide solution [20].

## 4. Mechanism of Action

In vitro studies revealed that hyaluronic acid beneficially controls cell migration and synthesis of intercellular substance. It improves cell migration speed, proliferation rate and an extracellular matrix production, emphasizing the significance of the cell microenvironment to wound healing process [27]. Depending on its molecular weight and presence of additional functional groups it can either stimulate or inhibit various metabolic pathways and processes in living organisms. HA stimulates the migration and proliferation of macrophages and smooth muscle cells and, hence, promotes a growth of atheroma. Moreover, it can boost tumour growth and metastasis by facilitating the infiltration of basal lamina and stimulation of angiogenesis [34].

Hyaluronic acid has the ability to interfere with inflammatory agents, such as prostaglandins and cytokines [30]. The overall effect of the regulation of the processes in living organisms by HA depends on its molecular weight. High molecular weight HA inhibits cell proliferation and exhibits an anti-oncogenic effect whereas HA of the medium molecular weight, consisting of 10 to 100 disaccharide units, stimulates cancer growth, considerably activates cytokines, and induces inflammatory processes. Low molecular weight HA (<50 kDa) as a diagnostic marker correlates with metastasis in breast cancer [35]. The smallest oligosaccharide fragments can act as pro-oncogenes as well as anti-oncogenes because on one hand they induce angiogenesis and on the other they can evoke apoptosis [36]. Small HA fragments can selectively induce apoptosis of the prostate and bladder cancer, reducing the cancer cells motility and inhibiting Akt signalling pathway. Tavianatou and co-workers [37] showed that HA-containing matrix could induce spreading of cancer but, simultaneously, small oligosaccharides inhibited that process.

Moreover, hyaluronic acid acts as a lubricant, enhancing the free post-surgical tendon movement without limiting the diffusion of nutrients and wastes [30].

Several possible mechanisms of action of nanosilver upon bacterial cells are considered. Nanoparticles can accumulate in bacterial cell membrane affecting its morphology and permeability, resulting in the annihilation [24,29]. Moreover, coupling Ag NPs with cationic biopolymers enhances their interaction with anionic cell membrane, and potentiates their antibacterial effect [32].

After penetrating the cells, Ag^+^ ions are generated from nanosilver. They produce reactive oxygen species, which are capable of inducing apoptosis and damaging organelles and biomolecules [24,38,39,40]. Furthermore, they impact on respiratory chain enzymes and nucleic acids, modulating intracellular signal transduction pathways [24]. Nanosilver may induce DNA damage directly through DNA binding and indirectly by binding to associated proteins, the mitotic spindle components or producing oxidative stress [41]. It can also modulate the downstream signal transduction pathways, such as Src, Akt and PI3K [42].

It was found that nanoparticles may inhibit growth of several microorganisms, including strains of *E. coli*, *P. aeruginosa* (also these drug resistant), *E. faecalis*, *S. aureus*, *S. pyogenes*, *Mycobacterium tuberculosis* and *P. larvae* [30,43,44,45]. A synergism of nanosilver and some antibiotics, such as chloramphenicol, was observed [46]. However, Gram-positive bacteria are generally considered more resistant to Ag NPs than Gram-negative ones due to the presence of the additional peptidoglycan layer in their cell wall, which limits the nanoparticles’ uptake, [30,32] even though there are studies which show opposite results [21,33]. This fact is nothing unexpected as some biopolymers can promote interactions between cell wall and Ag NPs, and enhance its effectiveness [32]. It is worth mentioning that wound dressings containing Ag^+^ ions show antibacterial effect even after bacteria form a biofilm [19,21].

It is speculated that silver nanoparticles lead to protein denaturation and proton pumps destruction following the interaction with sulphur and phosphoric groups present on the fungal cell wall surface. Moreover, they most probably increase the permeability of the cell membrane, leading to its disintegration, ion equilibrium loss, metabolism, and respiration blocking, finally resulting in the cell death. Additionally, the role of oxygen free radicals is also considered [47,48]. It is, however, worth mentioning that due to a thicker cell wall, the antifungal effect of Ag NPs is delayed, compared to their action on bacterial cells [49]. Anti-inflammatory properties of nanosilver are manifested as reduction in the liberation of cytokines and metalloproteinases, decreasing the influx of lymphocytes and mastocytes, as well as apoptosis induction of inflammatory cells, which play an important role in the wound healing process and autoimmune disorders [30,50]. It was reported that the presence of silver ions reduces MMP activity both in vitro and in vivo. The possible explanation is that the zinc ion from the proteinase is replaced by the silver cation, which impairs the proteolytic activity of the enzyme [21]. Moreover, silver nanocarriers are recognized as ROS scavengers [29].

Furthermore, the anticancer properties of nanosilver can be explained by the mitochondrial damage and the release of the reactive oxygen species, leading to a DNA damage, a disruption of the cell membrane, an impairing of the normal cells development and an induction of apoptosis [51].

It is suggested that the antimicrobial effect of nanosilver depends on Ag ions release rather than is caused by the particle itself. This sets a challenge for the researchers to find the nanocomposites which would achieve optimal functionality [20,23].

## 5. Applications

Bactericidal, fungicidal, antiviral, anti-inflammatory, anti-angiogenic and anticancer properties make silver nanoparticles highly appreciated in medicine. They can also be useful in drug delivery, gene therapy, and as biosensors. Hippocrates used to cure wounds and ulcers with the silver powder [52]. Credé in the fall of XIX century was the first who applied silver nitrate in curing gonococcal eye inflammation in infants [53]. Before antibiotics were discovered, syphilis and tonsillitis had been also cured with silver [54].

It is widely known that silver nanoparticles can effectively inhibit the growth of both Gram-positive and Gram-negative bacteria. Their efficacy in the complexes with biopolymers seems to be determined by the metal concentration [27,32,55]. Nanosilver prepared using either hyaluronic acid or heparin exhibited bactericidal properties against *S. aureus* and *E. coli*. What’s surprising, the material containing hyaluronic acid showed stronger antibacterial activity compared to those synthesized using heparin, possibly due to rougher surfaces, providing higher surface area exposed to the environment [56]. In the case of Methicillin-resistant *Staphylococcus aureus* minimal inhibitory concentration determined for the combination of allicin and nanosilver was considerably lower than that for both components separately [57]. The sphere-shaped Ag NPs present strong antibacterial activity and biocompatibility, which qualifies them for the treatment of bactericidal keratitis [58].

Moreover, nanosilver exhibits a broad spectrum of antifungal activity. In the combination with fungicides, such as amphotericin B and fluconazole, the effectiveness increases [59,60].

It is still discussed whether Ag NPs can be applied in suppressing viruses. According to the recent literature it is active against *Influenza A virus* [61], *respiratory syncytial virus* (RSV) [62], *Herpes simplex virus* type 1 (HSV-1) [63] and *Hepatitis B virus* (HBV) [64].

Various studies suggest that silver nanoparticles demonstrate anti-angiogenic properties and may be useful in the treatment of angiogenesis-related diseases, such as cancer, inflammation, and vascular diseases. Biosynthesized silver nanoparticles reduced the expression of the vascular endothelial growth factor (VEGF) and its receptor (VEGF-R) genes, and significantly reduced the number of vessels and their length, as well as the length and weight of the embryos in chick chorioallantoic membrane (CAM) assay [65].

Numerous in vitro and in vivo studies showed anticancer activity of nanosilver in various cancer cell lines, such as leukaemia THP-1, Jurkat, and K562 cells [66], MCF-7 [65] and MDA-MB-231 breast cancer cells [67], HepG2 liver carcinoma cells [68], nasopharyngeal cancer [69], HT144 melanoma cells, lung cancer, and retinoblastoma (RB), for which Ag NPs turned out to be a promising agent, especially due to their enhanced ocular targeting capability [70]. As a part of the combination therapy, they may be useful for efficient modulation of cancer surviving mechanisms, starvation therapy, combating multidrug resistance and to mitigate the drug side effects [71,72].

Silver nanoparticles can be also used in synergy with hyaluronic acid and S-nitrosothiol as the tumour targeting drug delivery system combined with photothermal therapy (PTT) and chemotherapy, increasing their selectivity [73,74]. What is more, due to the presence of the receptors for hyaluronic acid in various cells and due to the unique properties of this polysaccharide, HA can be used in cell- or tissue-targeted drug delivery systems (for nucleic acids or antitumor drugs), and for continuous release of protein drugs [75,76]. Hyaluronic acid used as gelling agent increased the efficacy of Ag NPs, probably due to the improvement of transportation mechanism across the cellular membrane and extended therapeutic activity time [77]. Lallana and co-workers [78] successfully delivered mRNA to the CD44 cells using chitosan/hyaluronic acid nanoparticles. The optimal performance of these nanoparticles was registered in slightly acidic pH, which is specific for the extracellular environment of tumour. Similarly, Martens et al. developed a novel retinal gene therapy, in which polymeric DNA complex-based nanomedicines were enveloped by the HA coating. It resulted in improved complex motility in the vitreous humour as well as efficient drug endocytosis through CD44 receptor [70]. Another example of the use of hyaluronic acid in drug delivery and release is given in the study of Liu and co-workers, describing inhalable hyaluronic acid microparticles loaded with hydrophobic budesonide nanocrystals that exhibited significantly prolonged pharmacological effect in vivo, as compared to inhaled budesonide nanocrystal suspension in rats [79].

Nanosilver can be employed in construction of catheters and tracheal tubes [80,81,82]. [83,84] Applicability of nanosilver in dental materials is under study [85,86]. Bandages and dressings containing nanosilver for curing burns [87], toxic epidermal necrolysis, Steven-Johnson syndrome [88] and pemphigus [89] are commercially available. Nanosilver can be also very useful in the prevention and the treatment of the diabetic foot syndrome [90,91]. Wound dressings containing the combination of nanosilver and hyaluronic acid become also highly hygroscopic, which enables the removal of excessive exudate from the wound beds [29]. They are also characterized by a good water-vapor transmission rate value (WVTR) and are therefore able to prevent the wound from becoming dry and dehydrated, ensuring proper environment for skin tissue regeneration [21].

**Figure 4:**
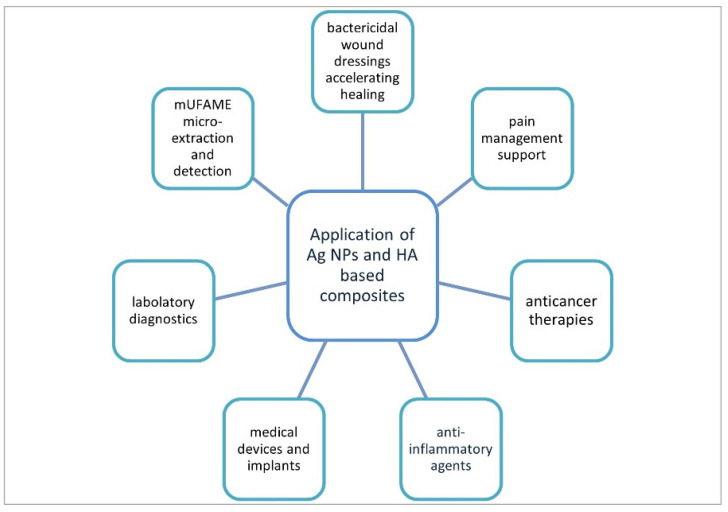
Potential application and usage of silver nanoparticles and hyaluronic acid-based composites; mUFAME—monounsaturated fatty acid methyl esters.

Potential applications and usage of AgNPs and HA compounds are shown in the Figure 4.

## 6. Cytotoxicity

Nanosilver is commonly used in many areas of the human everyday life, for instance, in textiles, deodorants, paints, packaging, and kitchen equipment. Such a wide use of silver evokes questions about its influence on human organism. In general, Ag ions are regarded to be cytocompatible at low concentrations. However, high doses of silver ions released in a short time may induce cytotoxicity [20].

Silver can be accumulated in the skin, generating its blue-grey colour—a primary symptom of argyria, caused by long-term exposition to silver, which has already been described in XVIII century [92]. Recent studies point out to the cytotoxicity of silver, among others, to keratinocytes [21], mononuclear leucocytes [93] and macrophages [94]. In vivo experiments show that nanosilver accumulates in tissues of laboratory rats resulting in toxic damages of their livers; the changes in other organs have not been, however, observed [95]. The cytotoxicity results mainly from the oxidative stress induced by Ag NPs, but in the case of nanoparticles larger than 10 nm also from the release of the Ag^+^ ions. A direct effect of nanoparticles on the mechanisms of cell regulation and gene expression can also be considered. In a recent study, Vazquez-Muñoz and co-workers [96] assessed the toxicity of the poly (vinylpyrrolidone)-coated silver nanoparticles, characterized by the average size of 35 nm, on various organisms: viruses, bacteria, microalgae, fungi, as well as on the animal and human cells, including cancer cell lines. They found that the growth of all tested biological systems was inhibited in vitro at the similar concentration of silver, close to 10 µg/mL. The obtained results may be explained by the fact that silver nanoparticles affect primitive cellular mechanisms and interact with the structures which are fundamental for all living cells.

However, the efficient stabilization of silver nanoparticles by the polymeric coating, and resulting low release of Ag^+^ ions, can minimize the toxic effect of such system on mammalian tissues [21]. The cytotoxicity depends on the size, shape, and chemistry of the coating [32]. Makvandi and co-workers [27] demonstrated that hydrogels containing Ag NPs, hyaluronic acid and corn silk extract demonstrated satisfactory biocompatibility. Furthermore, their application resulted in a better wound closure and lower wound area compared to the control samples in wound healing assay. These results are consistent with the results reported by Francesco and co-workers, [32] who did not observe any significant changes in the viability and metabolism of human skin fibroblasts and keratinocytes after one week of incubation with composite films obtained by sequential deposition of biopolymer (chitosan or aminocellulose)-capped Ag NPs and HA.

## 7. Examples of Usage

The hyaluronidase-triggered nanosilver release was investigated by Ran and co-workers [97], who obtained a photothermal nanocomposite of hyaluronic acid coated silver nanoparticles, which were next integrated within the graphene oxide. The composite presented low cytotoxicity to human cells and provided excellent antibacterial activity against *S. aureus*. The selectivity was explained by the ability of the bacteria to secret hyaluronidase, which degraded the hyaluronic acid coating, resulting in the release of the nanosilver. What is more, upon illumination with the near-infrared light, the temperature of the graphene oxide increased locally, which led to the high mortality of bacteria.

Guanqgian Lan and co-workers [98] prepared the sponges composed of chitosan, L-glutamic acid, hyaluronic acid and nanosilver. These sponges inhibited the growth of *S. aureus* and *E. coli*, while, at low concentration, they were not toxic to the L929 fibroblast cells. The in vivo test with rabbits and histological studies delivered evidence that these composites accelerate wound healing. A composite of κ-carrageen, hyaluronic acid, nanosilver and lidocaine also cured chronic ulcerations [99] indicating lack of cytotoxicity to human cells alongside bactericidal properties. Simultaneously, it was a good carrier for lidocaine. Thus, that material acts also as a pain reliever.

Properties of a titanium material covered with multilayer coat made of chitosan, hyaluronic acid and nanosilver were suitable for bone and dental implants [100]. In the first four days it fully protected against planktonic and fixed forms of *S. aureus*, and after 14 days its efficiency declined to 65–90%. That efficiency depended on a concentration of released nanosilver particles, but only for the first 14 days; then this correlation was no longer observed. Cytotoxic effect of the composites on MC3T3 mice osteoblasts also depended on the concentration of nanosilver.

Foils prepared, in a free of toxic reagents manner, from silver nitrate, xylose, hyaluronic acid and lecithin are promising dressings for healing wounds [26]. It was shown that lecithin has neither negative nor positive effect on bactericidal properties of those foils against *S. aureus, S. epidermidis* and *E. coli*. The molecular weight of hyaluronic acid increased considerably when Ag NPs were formed within its structure. After admixing lecithin, the material was characterized by a more uniform profile of molecular weight, which was considerably higher than that in the lecithin—free nanocomposite, pointing out to the possibility of formation of the intermolecular complexes. Lecithin containing foils were more flexible and mechanically more resistant.

Abdel Mohsen and co-workers prepared hyaluronic acid—nanosilver nanofibers and then built them into the nanosheets [101]. Fibres made of pure hyaluronic acid presented some bactericidal properties against *E. coli K12,* but an admixture of nanosilver considerably enhanced that effect. It was shown in vitro on the human keratinocyte HaCaT cell line, that neither hyaluronic acid alone nor its composites with nanosilver were cytotoxic. Studies on mice indicated positive effect of those composites against chronic ulcerations.

Sponges from polygalacturonic acid, hyaluronic acid and nanosilver were shown to exhibit bactericidal properties in vitro against *B.* *Subtilis, S. Aureus* and *E. Coli* [29].

Composites based on Ag NPs and HA were also proposed as components of the systems for detection of marker substances, thus allowing for the early recognition of neoplasms. Yang and co-workers [102] exploited affinity of HA to CD44 receptor and prepared a nanoprobe, allowing to test urine samples for the presence of bladder cancer cells. Au-Ag particles were involved in the reception of signals in the SERS method and were proven to provide an efficiency similar to selective antibodies in the detection of markers such as EGFR receptor, transferrin receptor, glycoprotein, folate receptor and CD44.

Other approach to implement biopolymers into the laboratory diagnostics was proposed by Li and coworkers [74]. Their work revealed that the system comprised of Ce-MoF (cesium-metal organic framework), HA, Ag NPs and horseradish peroxidase (HRP) may be a useful tool for detection of carcinoembryonic antigen (CEA) with high sensitivity.

**Figure 5:**
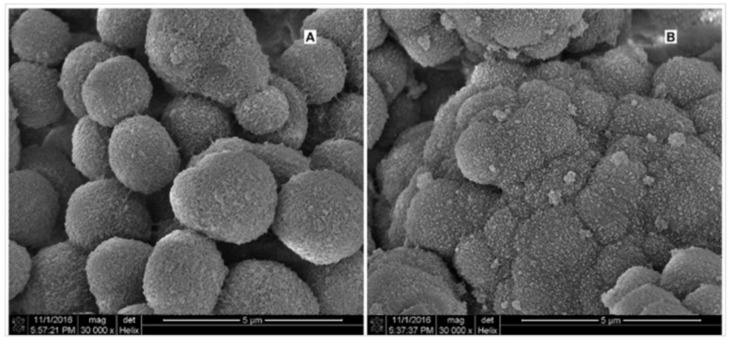
SEM micrographs of parent HA-UF monolith (**A**) and Ag NPs-coated HA-UF monolith (**B**). Reprinted from Reference [25] with permission from Elsevier.

The properties and potential biomedical applications of selected, important nanocomposites containing nanosilver and hyaluronic acid proposed in the literature are summarised in Table 1. SEM image of the polymer (sodium hyaluronate-functionalized urea-formaldehyde; HA-UF) coated with silver nanoparticles is depicted in Figure 5.

## 8. Challenges and Opportunities

Despite many favorable biochemical properties of Ag NPs-HA biocomposites there are a few concerns to be solved. First of all, the nanocomposites still show cytotoxicity on the adjacent healing tissue, which was discussed previously. The method of lowering the amount of released silver ions and nanoparticles would be desirable, nevertheless currently achieved profile is acceptable and safe. The second challenge is the synthesis process. Although the synthesis of membranes and even hydrogels serving as wound dressings is not complicated, production of biosensors and drug transporter systems using HA and AgNPs still remains a challenge in terms of technology and costs. Synthesis pathways simplifying the fabrication process would positively influence the availability of composites and potentially broaden their applications. The development of green-chemistry approach to the biopolymer synthesis is also a promising way of cost reduction and in our opinion might be the main target of the future research in this field.

## 9. Conclusions

The application of hyaluronic acid-nanosilver composites in medicine has an innovative character. Their bactericidal, antiangiogenic, and anticancer properties, as well as low cytotoxicity to human cells, encourage researchers and clinicians to seek broader application, predominantly as innovative wound dressings with antimicrobial capacity against drug-resistant microorganisms, accompanied by analgesic and healing-supportive features. Moreover, the composites appear to be promising components of oncological diagnostics and treatment. In our study we also introduced the materials that demonstrated satisfactory mechanical properties as potential medical implants designed for dentistry and orthodontics. The main advantages of hyaluronic acid-containing complexes over other silver nanocomposites include their high biodegradability and green synthesis, without use of any toxic reagents.

Take home message:

∎ Hyaluronic acid-nanosilver composites exhibit antimicrobial, anti-angiogenic, anti-inflammatory and anticancer properties, accompanied by a high biocompatibility.
∎ There are various techniques of synthesis of nanocomposites affecting their physicochemical properties, but the crucial points of the majority of the procedures include obtainment of the AgNPs and incorporation of them into biopolymer structure.
∎ Their potential applications include wound dressing production, oncological, vascular and inflammatory diseases treatment, as well as medical devices and implant fabrication.

## Figures and Tables

**Table 1:** Properties and potential applications of Ag NPs and HA containing composites.

Nanocomposite	Properties	Potential Applications	References
GO-HA-AgNPs	antibacterial against *S. aureus*, low toxicity to mammalian cells	diagnosis and treatment of bacterial infections	[97]
HA-Ag (nanofibers, nanosheets)	good mechanical properties, bactericidal against *E. coli* K12, nontoxic to human keratinocyte cells HaCaT; positive effect on chronic ulcerations	wound and chronic ulcer treatment	[101]
Chitosan/L-GA/HA/AgNPs (sponges)	good mechanical properties, swelling, water retention capacity, inhibition of *S. aureus* and *E. coli*; nontoxic to L929 fibroblast cells at low concentrations, promotion of wound healing	antibacterial wound dressings	[98]
CARR, HA, LID, AgNPs freeze-dried wafers	good porosity, bactericidal against *E. coli*, *P. aeruginosa* and *S. aureus*, nontoxic to human keratinocyte cells, fast release of lidocaine	treatment of pain in chronic leg ulcers	[99]
Ti-PTL-HA-CS/Ag	protection against planktonic and fixed forms of *S. aureus*, concentration-dependent silver cytotoxicity on MC3T3 mice osteoblasts	medical devices—catheters, wound dressings, bone cement	[100]
Hyal/Ag, Hyal-L/Ag (foils)	elasticity, water solubility, bactericidal against *S. aureus*, *S. epidermidis* and *E. coli*	prophylaxis and therapy	[26]
(Ag-PGA/HA) -PVA nanofibers	effective against gram-positive bacterial strains; Bacillus subtilis and *S. aureus*, as well as Gram-negative bacterial strain; *E. coli*	quick healing of wound infection	[29]
CSNMs with a PEG/PCL/Ag shell (PPA) and HA core	provides lubrication effect and reduced fibroblast attachment, bactericidal and anti-inflammatory properties,	management of post-surgical tendon adhesion	[30]
AgNPs-HA coated HA-UF monolith	satisfactory extraction efficiency towards unsaturated compounds	microextraction of monounsaturated fatty acid methyl esters	[25]
CH-PUL-HA/CS with AgNPs	good propensity to promote fibroblast proliferation, good biocompatibility, antimicrobial properties against *S. aureus*	treatment of chronic wounds (venous leg ulcers, diabetic foot, bed sores, burns and surgical lesions)	[24]
PGA-HA with AgNPs	antioxidative and anti-inflammatory, antibacterial against Gram + and Gram—bacteria, hydrophilicity,	quick healing of wound infections	[29]
Chi@Ag NPs/HA composite coating	bactericidal: sensitivity to bacteria secreting hyaluronidase for the controlled delivery of Ag ions, a good effect on the inhibition of bacterial growth	antibacterial surface with a controlled release of Ag ions	[20]
HA-Ag@S- nitrosothiols core-shell nanoparticles	“light-to-heat” transformation ability, first successful synergistic tumour targeting therapy performance	chemo- and photothermal synergistic tumor target therapy	[73]
Ce-MoF-HA-AgNPs-HRP	a broad linear response range, good reproducibility, selectivity, stability, without matrix effect	sensitive detection of carcinoembryonic antigen (CEA)	[74]
HA-MAgNPs	anticancer, fluorescence and photoactive properties	local treatment of cancer	[77]

## Data Availability

Data sharing not applicable. No new data were created or analyzed in this study. Data sharing is not applicable to this article.
